# Bromelain: A Potent Phytomedicine

**DOI:** 10.7759/cureus.27876

**Published:** 2022-08-11

**Authors:** Paridhi Agrawal, Pradnya Nikhade, Aditya Patel, Nikhil Mankar, Shweta Sedani

**Affiliations:** 1 Department of Conservative Dentistry and Endodontics, Sharad Pawar Dental College, Datta Meghe Institute of Medical Sciences, Sawangi, Wardha, IND; 2 Department of Conservative Dentistry and Endodontics, Sharad Pawar Dental College and Hospital, Datta Meghe Institute of Medical Sciences, Wardha, IND; 3 Conservative Dentistry and Endodontics, Sharad Pawar Dental College and Hospital, Datta Meghe Institude of Medical Sciences, Wardha, IND

**Keywords:** antimicrobial, bromelain enzyme, bioactive, phytomedicine, bromelain

## Abstract

The commercially available protein-digesting enzyme bromelain is derived from the pineapple fruit or stem. Bromelain from fruit and stems are produced in different ways and has varied enzyme compositions. “Bromelain” often refers to the "stem bromelain". Bromelain is a combination of several thiol endopeptidases and others including various protease inhibitors, glucosidase, cellulase, phosphatase, peroxidase, and escharase. Studies conducted in both the lab and on animals show that bromelain has a variety of fibrinolytic, anti-edematous, antithrombotic, and anti-inflammatory effects. The body can absorb bromelain to a significant extent without it ceasing its proteolytic activity or having any negative side effects. Numerous therapeutic advantages of bromelain include wound debridement, improved drug absorption, and the management of sinusitis, bronchitis, angina pectoris, surgical trauma, and thrombophlebitis. Additionally, it treats numerous cardiovascular conditions, diarrhoea, and osteoarthritis. Bromelain also encourages apoptotic cell death and exhibits some anti-cancerous properties. This review compiles the crucial traits, medical and dental uses of bromelain as well as its potential mechanism of action.

## Introduction and background

Infectious diseases, wherein antibiotics are often used as first-line therapy to treat bacterial infections, are among the greatest threats to human health globally [1]. Nonetheless, extensive usage and imprudent intake in animals and humans have shot up the resistant bacterial strains. Furthermore, one microbe can develop resistance to multiple antibiotics, culminating in multidrug resistance strains for a range of microorganisms, some of which include Enterococcus faecium, Staphylococcus aureus, Pseudomonas aeruginosa, Salmonella species, Campylobacter, Neisseria gonorrhoeae, Streptococcus pneumonia [2]. Corollary to this, the expense of hospitalization and healthcare, along with morbidity and mortality, are sky shooting [3]. In response, it is crucial to seek out new therapeutic candidates and innovative alternative therapies [4]. When natural antimicrobial proteins were used as therapeutics, numerous studies demonstrated promising results [5], notably because their use in conjunction with traditional chemotherapeutic agents enhances efficacy, reduces antibiotic use, and presumably diminishes incidents of chemoresistance [6]. Consequently, natural therapeutic medicines derived from medicinal plants have gained recent popularity [7] due to their lack of side effects, non-toxicity, affordability, and accessibility [1].

It has been established that the pineapple plant, in particular, possesses therapeutic characteristics. Despite the presence of additional proteolytic enzymes in the pineapple plant, such as comosain and ananain [8], bromelain is the most studied [9]. It belongs to the Bromeliaceae family [10]. This proteolytic enzyme (protease) catalyzes the breakdown of proteins into amino acids [10-13]. It was discovered in 1891 [10], but it took a long time to thoroughly isolate, purify, and describe it [11,14,15]. Bromelain's usage as a synergistic agent with conventional treatment has been extensively documented in clinical studies [16]. It has ubiquitous nature and is used as a phytomedicine [17]. Bromelain possesses great therapeutic potential with a plethora of proteinase inhibitors [18]. Bromelain is a predominant oxidizing agent [19]. Its fibrinolytic, proteolytic, antifungal, antibacterial, antithrombotic, and anti-inflammatory effects are well established [10]. It also has anti-cancer functions [18]. This defense protein bromelain also protects the pineapple plants during their development, maturity, and ripening processes [19,20]. This review sweepingly altercates the source, structure, biochemistry, biogenesis, immobilization strategies, bioavailability, therapeutic applications, synergistic effects, side effects, toxicity, and future perspectives of bromelain. The phrases “bromelain”, “bioactive”, “antimicrobial”, “anticancer”, “anti-inflammatory”, “toxicity”, “immobilization”, “adsorption”, and “carrier” were used to retrieve this report from PubMed, Web of Science and Scopus databases. The objective of this review article is to spotlight the characteristics of bromelain that make it an ideal candidate for dental applications. In this context, we also refer to the fact that there have been very few clinical trials utilizing bromelain, which is in stark contrast to their potential in the medical and dental sector. As ostensively mentioned, in future perspective, the possibility to use bromelain as a direct pulp capping agent or an intracanal medicament has been pushed.

## Review

Source of bromelain

Vicente Marcano, a Venezuelan scientist, discovered bromelain for the very first time in 1891, and its extraction and investigation commenced in 1894. Bromelain is abundant in both the fruit and the stem of pineapple trees, with Heinecke revealing in 1957 that the pineapple stem contained significantly more bromelain than the actual fruit [21], enabling the monetization of a waste by-product that is stem bromelain [19]. Crude pineapple aqueous extract is used to purify its defensive protein bromelain [1]. This protein of the pineapple plant shields it during its growth, maturation, and ripening periods [20,22]. Bromelain is extracted as a glycosylated monomeric single protein from both the stem and the fruit [23]. The differences between stem and fruit bromelain are mentioned in Table 1 [1,20,21,24-26].

**Table 1:** Differences between stem bromelain and fruit bromelain EC- Enzyme Commission Number

	Stem bromelain (EC 3.4.22.32)	Fruit bromelain (EC 3.4.22.33)
Source	Pineapple stem	Pineapple fruit
Molecular weight (kDa)	23.8-37.0 [24]	23.0-32.5 [24]
Isoelectric point	>9.5 (Alkaline protein) [21]	4.6 (Acidic protein) [21]
Amino acid sequence	212, 291, 285 [25, 26]	326,351 [25, 26]
Optimum Temperature [Celcius]	40-60	37-70
Optimum pH	4-8	3-8
Presence of Glycoproteins	Yes	Yes/No

Structure and biochemistry of bromelain

Bromelain is a protease that catalyzes proteolytic processes [12,20,27], and it is categorized as a cysteine proteinase (EC 3.4.22, CP, commonly known as thiol proteinase) because its active site has cysteine thiol [1,20]. Several cysteine endopeptidases (pineapple fruit- fruit bromelain, pineapple stem - ananain, stem bromelain, comosain) and other elements, such as phosphatases, peroxidases, carbohydrates, ribonucleases, protease inhibitors, cellulases, glycoproteins, and organically bound calcium are present in crude bromelain [1,13,14,21]. A sulfhydryl moiety makes up its functional element [16]. Bromelain from the stem has a stable secondary structure. In between pH 7 and 10 it is active, but it loses its action irreversibly above pH 10 [28]. At a pH of 14, stem bromelain forms a typical heated gelatinous mass configuration [29]. Finally, bromelain has been shown to remain stable for a long time when kept at temperatures below 20 ºC [30].

Biogenesis of bromelain

All components of the pineapple plant (stem, core, peel, crown, and leaves) can be purified to obtain bromelain with a difference in concentration and composition. Pineapple stem and fruit yield high titres of bromelain when compared to pineapple core, peel, and leaves, but along with pineapple stem and crown, they reach up to 50% (w/w) of the cumulative pineapple waste [27], making bromelain isolation from pineapple waste economic and ecologic [31]. As a consequence, the most widely viable bromelain originates from the pineapple stem, which is also much more therapeutically efficacious and has more proteolytic activity in comparison to fruit bromelain [12].

Immobilization of bromelain

One of the problems with using enzymes (like bromelain) is that their activity decreases after processing or over time [32]. Immobilization is a potential method for ensuring their efficiency [33]. The interactions between the carrier and the enzyme must be thoroughly understood and controlled for successful immobilization [34]. Few immobilization strategies for bromelain could be entrapment into hydrogels, adsorption onto chitosan matrix, covalent immobilization, and entrapment into nanoparticles. Ataide et al. [35], in a study, concluded that the nanoencapsulation technique allows for sustained bromelain release, resulting in antiproliferative and antioxidant benefits, depending on the duration of exposure. Also, bromelain nanoencapsulation with chitosan offered physical protection, wound retraction, and delayed release, all of which are desirable in topical formulations with a modified release. It is possible to reduce the carrier's influence on the structural as well as functional properties of bromelain, strengthen its stability and activity when exposed to high temperatures and a wide pH range, and improve its anti-inflammatory and antimicrobial activity by choosing a suitable carrier and immobilization method [34]. However, for bromelain transfer, there is currently no standard, highly efficient immobilization method [36].

Bioavailability of bromelain

Animal pharmacokinetic investigations have shown that the enzyme complex is absorbed in a rate of roughly 40% via the enteral route, it shows peak concentrations in blood and has a short half-life [16]. This efficient absorption occurs due to its capacity to bind to the two main blood antiproteases, alpha1-antichymotrypsin, and alpha 2-macroglobulin. In an in vivo investigation, it was discovered that over 12 g/day of bromelain can be consumed without any noticeable negative effects [37]. Bromelain's plasma proteolytic action is also preserved [38]. Based on this, a recent in vitro study found that almost 30% and 20% of bromelain was stable in synthetic stomach juice and blood after four hours, respectively [39].

Antimicrobial efficacy of bromelain

Chemical constituents, such as tannins, saponins, flavonoids, and several enzymes, present in bromelain exert antibacterial characteristics to it. Flavonoid has the inherited ability to form a complex link with the extracellular protein by hydrogen bonding, hence altering cell membrane permeability [10]. Bromelain's antiadhesion capability has been hypothesised to prevent bacteria from adhering to surfaces, resulting in antibacterial action [17]. Its efficacy in the treatment of infections caused by Vibrio cholera, helminthic, and Escherichia coli also, chronic inflammatory diseases such as Crohn’s disease and ulcerative colitis, urinary tract infections, sinus infections, and prostatitis has been demonstrated [16].

An in-vitro study by Chandwani et al. [10] evaluated and compared the antimicrobial efficacy of triple antibiotic paste and calcium hydroxide with Bromelain against Enterococcus faecalis the primary organism seen in refractory endodontic infections. They found that the optical density reading of Bromelain paste (0.495) was less than calcium hydroxide (0.557) and the optical density of Bromelain and triple antibiotic paste (0.441) were comparable. They concluded that antibacterial efficacy of bromelain was more than calcium hydroxide paste and comparable to triple antibiotic paste against E. faecalis [10]. Ali et al. in their study found that bromelain, in its crude form, may be a potent antibiotic against E. coli and Proteus species [40]. Bromelain is reported to show more effect against Gram-positive bacteria than Gram-negative bacteria [10,40]. Praveen et al. [17] in an in-vitro study calculated the minimum inhibitory concentration of bromelain on both anaerobic and aerobic periodontal pathogens and evaluated its antibacterial efficacy. They found that among aerobic bacteria, Streptococcus mutans produced sensitivity at 2 mg/mL in comparison to E. faecalis (31.25 mg/mL) while among anaerobic bacteria sensitivity shown by Porphyromonas gingivalis was least, at concentration of 4.15 mg/mL in comparison to Aggregatibacter actinomycetemcomitans (16.6 mg/mL). Bromelain may be employed as an antibacterial agent because, according to their findings, it has an antibacterial response against potent periodontal infections.

Therapeutic applications of bromelain

It is used to treat burns, allergies, blood coagulation, inflammation, antibiotic enhancement, blocked sinuses, cardiovascular disorders, osteoarthritis, diarrhea, and cancer, among other problems [10]. The aggregation of platelets both in vitro and in endothelial cells is prevented by bromelain. It also works as a fibrinolytic agent because it promotes the conversion of plasminogen to plasmin [16]. It also increases antibiotic absorption, resulting in effective drug diffusion in tissues and, as a result, a reduction in the probability of toxicity-related adverse effects [10]. It is regarded as a potent anti-edematous and anti-inflammatory agent that works on a variety of targets that respond to inflammation, including immune system cells, and the coagulation cascade [16]. Bromelain acts on the immune system by enhancing the production of tumor necrosis factors, interferons, granulocyte-macrophage colony-stimulating factors (GMCSF), and interleukins (IL-1, IL-2, IL-6). CD4+ helper T-lymphocytes activation is limited by bromelain thereby decreasing CD25 and CD44 expression. It stimulates p53 and Bax expression while inhibiting IB, Akt, extracellular regulated protein kinase (ERK) 1, nuclear factor-B (NF-B), and 2, p38 mitogen-activated protein kinase (MAPK). Bromelain may serve as an anti-inflammatory agent by decreasing bradykinin production in the Kinin-Kalli-Krein pathway, as well as suppressing leukocyte migration and adhesion through an antagonistic impact on CD128 receptors [16].

A study conducted by Hong et al. [41] to investigate bromelain’s mineralization and anti-inflammatory effects on lipopolysaccharide-induced human dental pulp cells showed that bromelain had no significant effect on the viability of human dental pulp cells at 2.5, 5, 10, or 20 microgram/mL. In human dental pulp cells, bromelain significantly decreased interleukin-1, 6, 8, ICAM-1, and VCAM-1 level which was induced by bacterial lipopolysaccharide. The cytoplasm and nuclear phosphorylation of p65 were considerably decreased by bromelain administration. Extracellular signal-related kinases and p38 mitogen-activated protein kinases' phosphorylation levels were also markedly reduced. ALP activity and formation of mineralized nodules were substantially elevated by bromelain. They concluded that in lipopolysaccharide-stimulated human dental pulp cells, the expression of anti-inflammatory cytokines was inhibited by bromelain. As a consequence, bromelain may have application in vital pulp therapy and regenerative endodontics [41]. This substance has been proven to function on many levels of the pathogenetic pathways of the acute inflammatory response in various studies. It has a synergistic action with antibiotics, oral anticoagulation therapy, and therapies for osteoarthritis, musculoskeletal injuries, and rheumatoid arthritis. Studies on its efficacy in the therapy of postoperative inflammation are also described in the literature [16]. Bromelain's efficacy in the prevention and treatment of cardiovascular disorders including angina pectoris, ischemia, and myocardial infarction, as well as musculoskeletal conditions like muscle injuries, osteoarthritis, and rheumatoid arthritis, has been proven. Finally, research has shown that the chemical is effective in lowering postoperative pain and edema. It has also been proved that there are almost no negative effects or overdoses [16]. Sehirli et al. [42] in their study found that in NaOH-induced corrosive burns, bromelain treatment reduced oxidative and inflammatory parameters while increasing antioxidant levels. Bromelain's anti-inflammatory and antioxidant properties were found to protect the esophagus and tongue tissues in corrosive burns. Ataide et al. [35] in their study concluded that bromelain produced antiproliferative effects against tumor cell lines. Bottega [43] concluded that bromelain modulates inflammatory indicators in human cell lines originating from the intestine, stomach, and chondrocytes. Also, following modeled gastrointestinal transit and digestion, anti-inflammatory effects were maintained. Chang et al. [44] found that bromelain supressed colorectal cancer cell growth. It also resulted in substantial quantities of superoxide, and reactive oxygen species, as well as the production of autophagosomes and lysosomes. Apoptosis was also triggered in high levels.

Dental applications of bromelain

After Third Molar Surgery for Improving Postoperative Course

Ordesi et al. [16] in their study evaluated bromelain’s efficacy to reduce postoperative pain and swelling after third molar surgery and compared it with routine analgesia groups. They concluded bromelain to be a safe and well-tolerated drug that, when used in conjunction with normal therapy, improves the postoperative course by reducing pain, erythema, and inflammation after extraction of the third molar. This study showed that bromelain had a predominant and statistically significant anti-inflammatory and anti-edematous effect in third molar surgery.

After Acid Etching to Improve the Bond Strength of Adhesive Systems

Before applying the adhesive system, Chauhan et al. [11] tested bromelain’s deproteinizing effect on shear bond strength and compared it with 5% sodium hypochlorite. They revealed that bromelain removes unsupported collagen fibrils following acid etching thereby reducing nanoleakage and improving bond strength. In comparison to the deproteinizing effect obtained with 5% sodium hypochlorite, results obtained with bromelain were greater and statistically significant. Dayem and Tameesh [45] found similar results in their study comparing bromelain’s deproteinizing effect to 10% sodium hypochlorite and Nd: YAG laser. They concluded that using the bromelain enzyme resulted in the removal of collagen networks and a considerable reduction in the global leakage scores (4.1) of the adhesive system, compared to the Nd: YAG laser (16.2) and 10% sodium hypochlorite groups (30.1). This may be related to the loss of collagen from acid-etched dentin's surface, which causes the dentin substrate's permeability to increase because of the enlargement of dentinal tubules which are closer to the outer surface of dentin. As a result, adhesive monomers diffuse and distribute more readily through dentin [46,47]. Because collagen has low surface energy whereas hydroxyapatite has a higher surface energy substrate, the dentin's surface energy is improved, which enhances the adhesive monomer’s diffusion through the dentin [45,48]. Additionally, the dentin is highly porous and rough, with numerous lateral tubule branches that can be seen in the major tubules, which may accelerate the diffusion of adhesive monomers across the dentin [49,50].

Reduction in Microleakage of Tooth-Colored Restorations

In an in-vitro study, Farahnaz and Paniz [51] evaluated the effectiveness of the deproteinizing characteristic of the 10% bromelain enzyme on the microleakage of composite and resin-modified glass ionomer cement restorations. They found that after using phosphoric acid and then treating with bromelain in the composite filling, microleakage on gingival and occlusal margins was significantly reduced whereas applying bromelain or polyacrylic acid had no effect on the microleakage of glass ionomer filling. They concluded that bromelain should be used in composite fillings after using phosphoric acid. They also suggested that bromelain should be used instead of polyacrylic acid in resin-modified glass ionomer cement restorations because of its anti-inflammatory properties. Bromelain efficiently removes collagen network from acid-etched dentin, increasing the monomer’s diffusion potential to the intact dentin and minimizing microleakage [51].

For Caries Removal

Bromelain enzyme acts on the pre-degraded collagen networks of the carious lesion, it softens them and promotes their easy removal. Reddy et al. [52] in an in-vitro study evaluated the efficacy of bromelain as the chemo-mechanical caries removal agent and concluded that it took 335 seconds for bromelain to remove caries and less amount of demineralized dentin was remaining thereafter.

For Dental Bleaching

The dental bleaching action is aided and accelerated by bromelain when used in conjugation with hydrogen peroxide. In their study, Vejai et al. [19] evaluated the color change in the human enamel that was bleached with hydrogen peroxide-containing pineapple extract as an additive, concluding that the groups using pineapple extract in addition to hydrogen peroxide showed statistically significant higher tooth lightening than the groups using only hydrogen peroxide. This is because the adherent proteins that cause dental stains are induced to split by the pineapple extract. It plays a crucial element in the bleaching process by weakening the protein component of the pellicle layer that adheres to the tooth surface. The pineapple decreases the activation energy of hydrogen peroxide (75 kJ/mol) and also improves the rate of chemical reaction efficacy by increasing the rate of free radical release. A complex with hydrogen peroxide is formed by the enzymes, increasing its bleaching efficiency while having less adverse effects on the enamel surface. Furthermore, as a result of this, the reaction time is expedited [19].

Bromelain and COVID-19

Tallei et al. [53] revealed that a fruit bromelain-derived peptide DYGAVNEVK interacts with several critical SARS-CoV-2 spike glycoprotein receptor-binding domain binding residues responsible for the adhesion of the receptor-binding domain to human angiotensin-converting enzyme 2 receptors and restrains its attachment. Akhter et al. [54] revealed that bromelain and acetylcysteine (BromAc) have synergistic action against glycoproteins of SARS-CoV-2 by breakage of its glycosidic linkages and disulfide bonds.

Synergistic effects of bromelain

Ali et al. [40] in their study concluded that the effect of combining crude bromelain with an antibiotic (Ampicloxacillin [2.5 mg/mL] + crude extract [0.9mg/mL]) was greater than either conventional or crude bromelain alone. Ghensi et al. [55] concluded from their study that dexamethasone sodium phosphate (20 ng/mL) when added to bromelain (20 ng/mL) activated a greater number of mesenchymal stem cells, resulting in increased hyaluronan and collagen synthesis as well as the release of anti-inflammatory cytokines. Lam et al. [56] found that bromelain and acetylcysteine (BromAc) is a newer therapeutic medication that has been shown to have mucolytic characteristics. It aids tumor dissociation and enables for swallowing when injected directly into mucinous illness. As previously mentioned, Akhter et al. [54] revealed that bromelain and acetylcysteine (BromAc) has synergistic action against glycoproteins of SARS-CoV-2 by breakage of its glycosidic linkages and disulfide bonds.

Toxicity of bromelain

The demand for pineapple fruit especially bromelain has gone through the roof around the world as a result of its health benefits and applications in a variety of fields [57]. Bromelain has very low toxicity, according to Taussig et al., with an LD50 (lethal doses) of more than 10 g/kg in rabbits, rats, and mice. After six months, toxicity testing on dogs with escalating levels of bromelain up to 750 mg/kg delivered daily revealed no harmful effects [58]. When rats were given doses of 1,500 mg/kg per day, there were no teratogenic or carcinogenic effects, and there were no changes in food intake, growth, kidney, heart histology, spleen, or hematological parameters [59]. Eckert et al. [60] reported that the parameter of blood coagulation did not change after providing bromelain (3,000 FIP unit/day) to humans for 10 days.

## Conclusions

Because of their advantages in maintaining human health and uses in a variety of contexts, bromelain and pineapple fruit demand has been rising significantly around the world. Because it is effective in the management of cancer, severe wounds, inflammation, osteoarthritis, dental plaque, gingivitis, and different infections, bromelain is regarded as a high-value enzyme in the therapeutics sector. Bromelain can be utilized as an alternative to numerous chemical components and synthetically produced medications because it is a natural and harmless substance. A protease enzyme, found in pineapples, is what separates other proteins by severing their amino acid chains. Bromelain indirectly intervenes to specifically block the formation of pro-inflammatory prostaglandins. It has been demonstrated that the endogenous protease plasmin and pineapple protease have identical sensitivity ranges.

It is hypothesized that bromelains cause additional inflammation of the arachidonate cascade at the thromboxane synthetase level. The major stages of COVID-19's pathophysiology are interfered with by bromelain and curcumin. Their ability to suppress transcription factors and thus reduce pro-inflammatory mediators is one of their anti-inflammatory capabilities. Additionally, bradykinin hydrolysis is inhibited by bromelain, along with the inhibition of cyclooxygenase, prostaglandins, thromboxane, inflammation, and coagulation. Future researchers should create more inventive extraction techniques to increase the market for bromelain because it has minimal toxicity and only moderate adverse effects. Adsorption-based separation and nanoparticle-based purification of bromelain are necessary. Research should be done to better understand bromelain's mode of action so that health professionals can avail advantage of its characteristics. Bromelain demonstrates multiple actions in the realm of pharmacology.
